# Book Reviews

**Published:** 1896-05

**Authors:** 


### Book Reviews.

---

A TEXT-BOOK UPON THE PATHOGENIC BACTERIA for Students of Medicine and Physicians. By JOSEPH MCFARLAND, M. D., Demonstrator of Pathological Histology and Lecturer on Bacteriology in the Medical Department of the University of Pennsylvania, etc. With One Hundred and Thirteen Illustrations. Philadelphia: W. B. Saunders, 1896. [Price, $2.50.]

This work, while intended as an elementary text-book for medical students, contains, as the author states in the preface, much of interest to the practitioner of medicine, especially to those who graduated before modern scientific research had thrown light upon the etiology of disease. The auther, by confining himself exclusively to Pathogenic Bacteria, has been enabled to keep the size of the book within bounds, and yet present clearly to the student not only the technique of bacteriology, but also to give a fair summary of what is at present known of pathogenic bacteria. In the chapter on immunity and susceptibility, the author has succeeded in compassing in twelve or fifteen pages, an excellent outline of what is at present known of this almost unentered field for investigation. Necessarily in so short a review, certain recent investigations have been omitted, but the chapter is entirely free from dogmatism or prejudice. The numerous photographic plates used throughout the text are, in the main, reproductions from standard works, and are as a whole good.

We take pleasure in commending the work to students and practitioners of medicine.

